# Environmental hotspots for antibiotic resistance genes

**DOI:** 10.1002/mbo3.1197

**Published:** 2021-05-15

**Authors:** Shalini Kunhikannan, Colleen J. Thomas, Ashley E. Franks, Sumana Mahadevaiah, Sumana Kumar, Steve Petrovski

**Affiliations:** ^1^ Department of Physiology, Anatomy and Microbiology School of Life Sciences College of Science, Health and Engineering La Trobe University Bundoora Vic Australia; ^2^ Department of Microbiology JSS Medical College and Hospital Mysuru India; ^3^ Department of Microbiology Faculty of Life Sciences JSS Academy of Higher Education and Research Mysuru India

**Keywords:** antibiotic resistance genes, antimicrobial resistance, clinical settings, environmental hotspots

## Abstract

Bacterial resistance toward broad‐spectrum antibiotics has become a major concern in recent years. The threat posed by the infectious bacteria and the pace with which resistance determinants are transmitted needs to be deciphered. Soil and water contain unique and diverse microbial communities as well as pools of naturally occurring antibiotics resistant genes. Overuse of antibiotics along with poor sanitary practices expose these indigenous microbial communities to antibiotic resistance genes from other bacteria and accelerate the process of acquisition and dissemination. Clinical settings, where most antibiotics are prescribed, are hypothesized to serve as a major hotspot. The predisposition of the surrounding environments to a pool of antibiotic‐resistant bacteria facilitates rapid antibiotic resistance among the indigenous microbiota in the soil, water, and clinical environments via horizontal gene transfer. This provides favorable conditions for the development of more multidrug‐resistant pathogens. Limitations in detecting gene transfer mechanisms have likely left us underestimating the role played by the surrounding environmental hotspots in the emergence of multidrug‐resistant bacteria. This review aims to identify the major drivers responsible for the spread of antibiotic resistance and hotspots responsible for the acquisition of antibiotic resistance genes.

## Graphical abstract

Antibiotic contamination or waste enters a variety of different environmental settings. The accumulation of antibiotics ​can cause a selective pressure on bacteria that encode resistance genes for their survival, generating a hotspot for resistance genes. These hotspots contribute to the spread of antibiotic resistance genes in the environment.

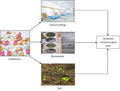

## 1 INTRODUCTION

Soil is an important rich source of nourishment for the survival of many microbial forms. Soil consists of four basic components namely, mineral particles (45%), water (25%), air (25%), and organic matter (5%) (Brady et al., 2008). The organic matter, in turn, contains humus (80%), roots (10%), and organisms (10%). Soil microorganisms include viruses, bacteria, actinomycetes, fungi, algae, and protozoa, and their prevalence is affected by soil temperature, moisture content, and available carbon sources. To gain an advantage in the soil environment many microbes have evolved survival mechanisms, such as producing antibiotics, to suppress competitors. A concomitant benefit is the suppression of other disease‐causing microorganisms. Indeed, soil microorganisms (Actinomycetes) are the source of most antibiotics used to treat humans today. The presence of these antibiotics in the soil has led to the development of antibiotic resistance mechanisms, both in bacteria that produce antibiotics and those that do not have this capacity (D'Costa et al., 2006).

The decades between the 1930s and 1960s were considered the golden era of antibiotics due to the number of antibiotics discovered (Nathan & Cars, 2014). Indeed, the discovery and use of antimicrobial agents in clinical practice are some of the great achievements in medicine, significantly increasing the life span of patients. However, the pace of discovery of new antibiotics has significantly slowed emerging resistant pathogens (Nathan, 2004). Moreover, in the 21st century, antibiotic resistance has evolved as one of the biggest public health threats (Munita & Arias, 2016). There are different drivers and “hotspots” contributing to the spread of antibiotic resistance in both household and clinical settings. In clinical settings, monitoring wards and hospital wastewater for antibiotic resistance are key sites of interest (Cacace et al., 2019; Karkman et al., 2018; Khan et al., 2019). However, no information currently exists regarding the spread of resistance from bacteria in the hospital to the environmental soil. The mechanism for bacteria developing resistance is acquired through gene mutation or horizontal gene transfer. This enables organisms to acquire resistance to a single antibiotic and being receptive to many mobile genetic elements. Moreover, organisms may acquire resistance to multiple antibiotics becoming multidrug‐resistant, resulting in difficulty treating patients in clinical environments (Munita & Arias, 2016). This review describes some of the major drivers responsible for the spread of antibiotic resistance and hotspots responsible for the acquisition of antibiotic resistance genes. It also briefs the possibilities of potential exchange of resistance genes between different soil bacteria, commensals, and clinical pathogens. Human pathogens capable of acquiring such resistance determinants are generating a challenge for the treatment of multidrug‐resistant infections.

## 2 THE DISCOVERY OF ANTIBIOTICS AND EMERGENCE OF ANTIBIOTIC RESISTANCE

Table 1 summarizes the different classes of antibiotics based on their origin and structure. It highlights the timeline of the discovery of different antibiotics based on the year they were first reported in the literature and when they were introduced clinically. The initial finding was the discovery in 1928 by Alexander Fleming, where the development of *Staphylococcus* bacteria was inhibited by a specific species of mold known as *Penicillium notatum*, which led to the discovery of the first “Modern Day” antibiotic, penicillin (Jeśman et al., 2011). It still took more than a decade to introduce penicillin for the treatment of bacterial infections. Before the idea of bacterial inhibition by secondary metabolites of mold, in 1921, Fleming observed that lysozymes in systemic fluids could dissolve bacteria.

**TABLE 1:** Different classes of antibiotics based on their origin and the timeline of discovery

Antibiotics from Actinomycetes				
Class	Discovery reported	Introduced clinically	Example	Source
Aminoglycosides	1944	1946	Kanamycin A	*Streptomycin kanamyceticus*
Tetracyclines	1948	1948	Tetracycline	*Streptomyces aureofaciens*
Amphenicols	1947	1949	Chloramphenicol	*Streptomyces venezuelae*
Macrolides	1952	1952	Erythromycin	*Saccharopolyspora erythraea*
Tuberactinomycins	1951	1953	Viomycin	*Streptomyces puniceus*
Glycopeptides	1954	1958	Vancomycin	*Amycolatopsis orientalis*
Lincosamides	1962	1963	Clindamycin	*Streptomyces lincolnensis* (Semi‐synthetic derivative of lincomycin)
Ansamycins	1959	1963	Rifamycin SV	*Amycolatopsis rifamycinica* (Semi‐synthetic derivative of lincomycin)
Cycloserines	1955	1964	Seromycin	*Streptomyces orchidaceus*
Streptogramins	1953	1965	Pristinamycin	*Streptomyces pristinaespiralis*
Phosphonates	1969	1971	Fosfomycin	*Streptomyces fradiae*
Carbapenems	1976	1985	Meropenem	*Streptomyces cattleya* (Synthetic molecule based on Thienamycin)
Lipopeptides	1987	2003	Daptomycin	*Streptomyces roseosporus*
Lipiarmycins	1975	2011	Fidaxomicin	*Dactylosporangium aurantiacum* subsp. *hamdenesis*

Antibiotics from other bacteria				
Class	Discovery reported	Introduced clinically	Example	Source
Polypeptides	1939	1941	Gramicidin A	*Bacillus brevis*
Bacitracin	1945	1948	Bacitracin A	*Bacillus subtilis*
Polymyxins	1950	1959	Colistin	*Paenibacillus polymyxa*
Mupirocin	1971	1985	Mupirocin	*Pseudomonas fluorescens*
Monobactams	1981	1986	Aztreonam	*Chromobacterium violaceum* Synthetic molecule based on SQ 26,180

Antibiotics from Fungi				
Class	Discovery reported	Introduced clinically	Example	Source
Penicillin	1929	1943	Amoxicillin	*Penicillium chrysogenum* Semi‐synthetic derivative of Penicillin
Fusidic acid	1958	1962	Fusidic acid	*Fusidium coccineum*
Enniatins	1953	1963	Fusafungine	*Fusarium lateritium*
Cephalosporins	1948	1964	Cefacetrile	*Acremonium chrysogenum* Semi‐synthetic derivative of Cephalosporin C
Pleuromutilins	1951	2007	Retapamulin	*Pleurotus mutilus* (Semi‐synthetic derivative of pleuromutilin)

Synthetic Antibiotics			
Class	Discovery reported	Introduced clinically	Example
Arsphenamines	1907	1910	Salvarsan
Sulfonamides	1932	1936	Mafenide
Salicylates	1902	1943	4‐ Aminosalicylic acid
Sulfones	1908	1945	Dapsone
Pyridinamides	1952	1952	Isoniaizid
Nitrofurans	1945	1953	Nitrofurantoin
Azoles	1959	1960	Metronidazole
Fluoro(quinolones)	1962	1962	Ciprofloxacin
Diaminopyrimidin	1950	1962	Trimethoprim
Ethambutol	1962	1962	Ethambutol
Thioamides	1956	1965	Ethionamide
Phenazines	1954	1969	Clofazimine
Oxazolidinones	1987	2000	Linezolid
Diarylquinolines	2004	2012	Bedaquiline

Adapted from Hutchings et al. (2019).

During the late 1930s, a group of synthetic drugs called sulfonamides became the first systematically used chemical substances for the prevention and treatment of bacterial infections and proved to be a revolution in antibiotic therapy. However, the success of these antibiotics was temporary. This is because the organisms that encountered the first commercially produced antibiotics gradually began to attain resistance to single antibiotics. Resistance to penicillin among *Staphylococcus*, specified by an enzyme (penicillinase) that degraded the antibiotic, is one of the most notable examples (Barber, 1947; Davies, 2012). The industry invested heavily in the production of antibiotic derivatives against the developed resistant strains (e.g., introducing methicillin, modified aminoglycosides, and other beta‐lactamase‐resistant penicillins and cephalosporins). Over time, however, bacteria developed ways to circumvent these antibiotics and this ended the so‐called golden era of antibiotics (Davies, 2012). Researchers in Japan were the first to report the transfer of multidrug resistance by elements referred to as “R factors” in 1959 (Macuch et al., 1967). Other international studies subsequently confirmed this mechanism (Kruse & Sørum, 1994; Linton et al., 1974). Efforts to prevent or eliminate horizontal gene transfer were unsuccessful (Davies, 2014). As resistance increased, most pharmaceutical companies closed down their antibiotic research and development programs, thus creating a void in the discovery of antibiotics (Harbarth et al., 2015). The last class of antibiotics approved for clinical use was the Diarylquinolines (e.g., Bedaquiline) in 2012. Salvarsan, introduced in 1910, is no longer used clinically. Fusafungin, a mixture of enniatins, was withdrawn from the market in February 2016 based on the recommendation of the European Medicines Agency (Hutchings et al., 2019). One of the current scientific challenges is to discover new active antibiotics against clinically relevant antibiotic‐resistant bacteria.

## 3 DRIVERS DISSEMINATING ANTIBIOTIC RESISTANCE GENES

**FIGURE 1:**
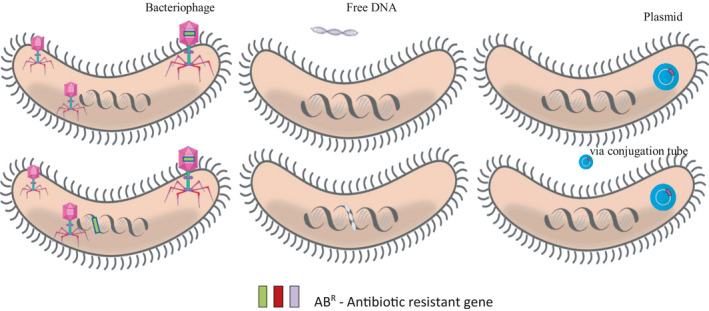
Acquisition of antibiotic resistance—the mechanism of horizontal gene transfer between different bacterial populations. There are three methods of transfer of genetic material: (1) transduction (via bacteriophage), (2) transformation (via free deoxyribonucleic acid (DNA)), and (3) conjugation (via plasmid). The antibiotic‐resistant gene becomes incorporated into the chromosome by recombination and/or transposition. Adapted from Alekshun and Levy (2007)

With extensive use of different drugs over time, microorganisms have emerged bearing additional kinds of resistance mechanisms leading to multidrug resistance. There are several eloquent reviews on this topic (Alekshun & Levy, 2007; Ayukekbong et al., 2017; Choudhury et al., 2012; Colodner et al., 2004; Gashaw et al., 2018; Nikaido, 2009; Tanwar et al., 2014). Alekshun and Levy (2007) highlight that these resistance mechanisms include novel penicillin‐binding proteins, enzymatic mechanisms of drug modification, mutated drug targets, enhanced efflux pump expression, and altered membrane permeability (Alekshun & Levy, 2007). Figure 1 illustrates that bacterial antibiotic resistance genes can be transferred through several horizontal gene transfer mechanisms.

Horizontal gene transfer (HGT) is one of the most widely known and prevalent processes among bacterial populations for the transfer of genes and enriching themselves with new characteristics or traits (Khan & Rao, 2019). HGT is a process of swapping genes between organisms that are not in a parent–offspring relationship (Soucy et al., 2015). This exchange of genetic material can cause both beneficial as well as adverse consequences. Although HGT plays a crucial role in biodiversity, innovations, and evolution (Jain et al., 2003; Ochman et al., 2000; Soucy et al., 2015), a recent review by Emamalipour et al. (2020) argued that HGT plays a role in the development of pathological conditions in case of disease (Emamalipour et al., 2020). Adding on to the significance of HGT, it plays a significant role in evolutionary genetics. It rescues prokaryotes from Muller's ratchet, an irreversible phenomenon, where the absence of recombination in asexual reproduction results in the accumulation of harmful mutations (Takeuchi et al., 2014; van Dijk et al., 2020). It is the primary mechanism for the spread of antibiotic resistance in bacteria and is achieved through the processes of conjugation (via plasmid and conjugative transposons), transduction (via bacteriophages), or transformation (via incorporation into the chromosome of chromosomal DNA, plasmid, and other naked DNA) (Levy & Marshall, 2004). It also plays a crucial role in the development of drug‐resistant microorganisms and the transfer of virulence genes. Gene transfer agents, first discovered in the 1970s (Solioz & Marrs, 1977), are another less common mode of gene transfer. It is a combination of bacteriophage transduction and natural transformation (Lang et al., 2012). They are small virus‐like particles responsible for the transfer of their entire genome between host cells (Solioz & Marrs, 1977). Among the three main mechanisms involved in HGT, transformation rarely occurs between bacterial species for the transfer of drug resistance genes. However, conjugation involving mobile genetic elements like transposons and plasmids is the most efficient and important method involved in the spread of antibiotic resistance (Price et al., 2019).

Gene transfer commonly occurs within the same genus, but it has also been observed between very different genera via the aforementioned methods (Evans et al., 2020; Jiang & Paul, 1998; Redondo‐Salvo et al., 2020). Lacroix and Citovsky (2016) have also reviewed the transfer of DNA from bacteria to Eukaryotes (Lacroix & Citovsky, 2016). For a gene transferred from another species to survive a long time in the recipient lineage, it is important to have a survival advantage either to the recipient or itself (Gogarten & Townsend, 2005).

Horizontal gene transfer is expedited by transposons and/or plasmids. These are the drivers responsible for the spread of antibiotic resistance (Khan & Rao, 2019). Microorganisms transfer resistance genes rapidly, spreading antibiotic resistance between the strains. The environment is considered to be the main source of mobile genetic elements, which are key elements responsible for the transfer of resistance genes (Knöppel et al., 2017). Recently, a study by Katale et al. (2020) revealed the presence of multiple sequence types isolated from the drug‐resistant bacteria and their replicon plasmid types from human, animal, and environmental sources. These findings highlighted a remarkable genetic diversity. Moreover, the presence of diverse antimicrobial resistance genes suggests an increased likelihood of multiple sources of resistant bacteria or a possible exchange of strains or gene flow among different strains due to transfer via mobile genetic elements (Katale et al., 2020). Next‐generation sequencing may be used as a better tool for the detection of evolving antibiotic resistance threats (Crofts et al., 2017). Performing whole genome sequencing on enteric bacteria, Kumar et al. (2017) confirmed the presence of multiple mobile genetic elements and horizontal gene transfer in six extensive enteric pathogens which were resistant to drugs (Kumar et al., 2017).

A deeper understanding of the significance of HGT has turned the focus of scientists to study the frequency of gene transfers. The frequency of gene transfers is of paramount importance, especially in the human microbiome. Although it is challenging to determine the frequency of HGT, an analysis that tried to explore gene transfers in the human microbiome revealed a surprising extend of gene transfer in human microbiota compared to other environments. The results showed a 25% greater frequency of HGT between pairs of organisms associated with humans than between pairs of organisms in different hosts or terrestrial or aquatic environments. Additionally, there was a 50‐fold likelihood of gene transfer between pairs of human‐associated organisms which were isolated from the same body site. This indicated that environmental fluctuations, which promote adaptive changes, were more prevalent in a holobiont ecology, especially human holobiont. The study highlighted that ecology governs HGT (Smillie et al., 2011).

One of the most important traits transferred via HGT is resistance to antibiotics. Antibiotic resistance can be achieved through intrinsic or acquired mechanisms. Intrinsic mechanisms are those specified by naturally occurring genes found on the host's chromosome and efflux systems. Acquired mechanisms involve gene mutations and/or transfer of resistance determinants via horizontal gene transfer. Such mutations in genes may cause resistance by (a) altering the target protein to which the antibacterial agent binds, modifying or eliminating the binding site; (b) upregulating the production of enzymes that inactivate the antimicrobial agent; (c) down‐regulating or altering an outer membrane protein channel that is required by the drug to bind and for cell entry, or (d) upregulating pumps that expel the drug from the cell. Despite a plethora of studies on the science behind resistance, limited information is available on the rise of resistant pathogens across the globe (Kumar et al., 2017).

## 4 HOTSPOTS FOR THE SPREAD OF ANTIBIOTIC RESISTANCE

Antibiotic‐resistant bacteria have been known to exist in the environment for many years. For example, there is evidence of their presence in caves up to 4 million years ago (Bhullar et al., 2012). Antibiotic‐resistant bacteria have been found in permafrost 30,000 years old (Finley et al., 2013), and they have also been detected in the gastrointestinal tract of individuals from Amazon tribes who have never been exposed to antibiotics (Gibbons, 2015). Certain bacteria are commensals and colonize people, however, they may cause disease when they traffic from their normal sites (e.g., skin and gastrointestinal tract) to areas of the body that they should not be (the bloodstream, organs, etc.). The drug‐resistant organisms and the antibiotics in the animal feed reach the environment through animal waste (Berendsen et al., 2015; Wichmann et al., 2014). The resistant organisms live close to each other in the soil, and this facilitates horizontal gene transfer through the transfer of genetic determinants (Christensen et al., 1998). In the case of humans, there is a definitive link between the consumption of antibiotics in animals and the development of antibiotic resistance in humans (O'Neill, 2016).

**FIGURE 2:**
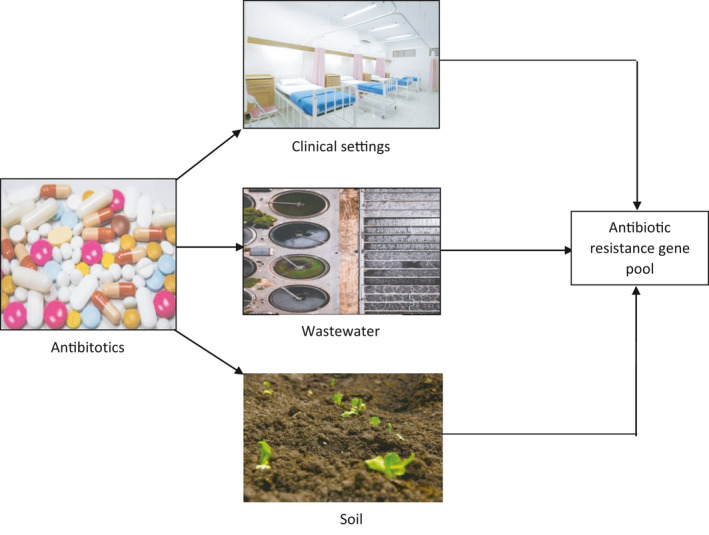
Antibiotic contamination or waste enters a variety of different environmental settings. Antibiotics are accumulated in these environments and select for bacteria that encode resistance genes for their survival generating a hotspot environment for resistance genes. These hotspots contribute to the spread of antibiotic resistance genes in the environment

Antibiotics are released into the environment via several pathways, such as clinical settings, human or animal waste, the use of products containing antibacterials, and through food and fodder which are supplemented with antibiotics and given to animals (Figure 2) (Gelband et al., 2015). They can even spread by contact with infected workers handling and processing meat or farm workers (Elbossaty, 2017; Michael et al., 2014). It is important to explore the different environmental hotspots facilitating the robust dissemination of resistance in pathogenic and non‐pathogenic bacteria. In this context, it is important to identify some of the major drivers which provide a favorable environment to hasten the process of antimicrobial resistance. Hotspots for antibiotic‐resistant bacteria include wastewater systems, pharmaceutical manufacturing sites, food and animal production sites in agriculture and aquaculture, and clinical settings such as hospitals (Berendonk et al., 2015).

### 4.1 Wastewater treatment plants

The selection of multi‐resistant bacteria and their spread into the surrounding environment from wastewater treatment plants is favored by the concomitant presence of antibiotics, heavy metals, antibiotic‐resistant bacteria, and antibiotic‐resistant genes (Di Cesare et al., 2016). Municipal wastewater systems serve as a hotspot for bacteria resistant to antibiotics (Berendonk et al., 2015; Di Cesare et al., 2016) and as a direct source responsible for the spread of resistance into the environment (Czekalski et al., 2014; Di Cesare et al., 2016; Gao et al., 2012; LaPara et al., 2011; Rizzo et al., 2013). Improper disposal of antibiotics and medical wastes from hospitals, leakage in septic systems, etc., facilitate the entry of antibiotic residues into the soil and groundwater. Though some antibiotics may degrade after reaching the environment, others can remain active for prolonged periods in wastewater treatment plants (Rizzo et al., 2013). High concentrations of antibiotics have been detected in water samples and sediments from the Liuxi River in Guangzhou, China, and fishponds are suggested to be the reservoirs of antibiotic residues and antibiotic‐resistant genes (Xiong et al., 2015). Xu et al. (2016) showed the occurrence and abundance of antibiotic‐resistant genes in drinking water treatment plants and distribution systems in China. Tap water supplied from these drinking water reservoirs to the residential areas contained resistance genes against some important classes of antibiotics considered useful in clinical practice (β lactams, aminoglycosides, and macrolide lincosamide streptogramin B) (Xu et al., 2016).

Studies have also reported the presence and dissemination of antibiotic‐resistant genes in environmental soil following the continuous release of reclaimed water irrigation containing antibiotic residues and antibiotic resistance genes (Wang et al., 2014). Poorly treated sewage also facilitates the transfer of resistant genes to human pathogens and the dissemination of antibiotic‐resistant bacteria (Bengtsson‐Palme et al., 2018). Even minute concentrations of antibiotics are sufficient to select for highly resistant bacteria, and this has been demonstrated in laboratory settings (Gullberg et al., 2011; Wistrand‐Yuen et al., 2018). As water‐containing antibiotics move through the sediments and soil, gradients of antibiotic concentration form.

### 4.2 Manufacturing industry

Antibiotic manufacturing industries release a significant concentration of antibiotics into the environment (Bengtsson‐Palme et al., 2018). Improper disposal of pharmaceutical wastes pollutes the natural environments like grasslands, water, and air (Sahoo et al., 2010). In Hyderabad, India, approximately 100 pharmaceutical manufacturing plants are involved in the supply of drugs to different parts of the world. However, a single plant processes the wastewater from all these manufacturing sites. Indicative of how easily drug contamination can occur following usage and incorrect disposal, Fick et al. (2009) previously reported in 2009 how processed effluent from the water treatment plant ended up in nearby surface water (lakes) as well as ground and drinking water (wells). These workers documented ciprofloxacin and cetirizine levels in the water which exceeded the human therapeutic concentration in blood plasma; indeed, the highest levels ever detected in surface and well waters at that time (Fick et al., 2009). The high levels of antibiotics in the water suggest likely mixing of water with the microbial population in the soil, creating a very favorable environment for the transfer of resistant genes between different species contributing to the spread of resistance (Gelband et al., 2015). A high number of multidrug‐resistant bacteria have been detected in treatment plants that harbor multiple bacteria from the environment, human pathogenic bacteria as well as normal commensal bacteria which do not cause any disease (Marathe et al., 2013).

### 4.3 Human and animal wastes – sanitary practice

Administering antibiotics to humans and animals results in antibiotics being excreted in the urine or feces, and thus, end up in the environment (Daghrir & Drogui, 2013; Sarmah et al., 2006). Additionally, animal feed containing antibiotics may directly seep into the soil or may be excreted through animals into the soil (Sarmah et al., 2006). It is known that wildlife represents a further reservoir of antibiotic‐resistant genes in the environment (Swift et al., 2019; Wellington et al., 2013). Animal waste and fertilizers (manure) can also be a source of resistant genes and bacteria entering the soil and groundwater (Sarmah et al., 2006). In the Netherlands, Berendsen et al. (2015) presented a multiclass method for the detection of around 20 classes of antibiotics in feces. Their analysis revealed that greater than 30% of collected pig and cattle feces contained the residue of at least one antibiotic. Three different antibiotics were recovered from pig samples while eight different classes were recovered from cattle feces (Berendsen et al., 2015). Such a study has not been conducted in a country like India, where it is common to see overcrowded shelter houses with more cattle and improper disposal of animal wastes. It is speculated that these livestock farms also serve as hotspots for the transfer of antibiotic resistance genes (Taneja & Sharma, 2019).

Smillie et al. (2011) identified bacteria from human food and farm animals and concluded that resistance transfers were more evident among human‐associated bacteria compared to non‐human‐associated bacteria. The study identified unique antibiotic‐resistant genes (belonging to the gene family—*aac*, *aph*, *catA*, *erm*, *sul*, *and tet*) transferred between human and farm isolates. The transferred genes comprised of nine gene families and targeted at least one antibiotic used in agriculture. Smillie et al. (2011) speculated that in the case of these mobile traits like antibiotic resistance, it is genes and not the genomes that serve as a unit of evolution and proliferation. Hence, livestock‐associated bacteria obtained from farm animals or human food, comprising of antibiotic resistance genes conferring resistance to both clinical antibiotics and agricultural drugs like amikacin, gentamycin B, kanamycin, lividomycin, neomycin, paromomycin, streptomycin, lincosamide, macrolide, streptogramin B, sulfonamide, and tetracycline can contribute to clinical resistance without infecting humans directly (Smillie et al., 2011).

### 4.4 Commensal bacteria

Commensal bacteria supply the host with essential nutrients and help protect the host from opportunistic pathogens; they are also important contributors to the pool of antibiotic resistance genes present in the surrounding environment. There is evidence for the transfer of antibiotic resistance genes between commensal bacteria and bacterial pathogens in the human intestine (Salyers et al., 2004). For example, fecal *E*. *coli* (commensal) has been universally acknowledged and explored as an indicator for the surveillance and spread of acquired antibiotic resistance genes among pathogens in community settings (Bartoloni et al., 2006; Lester et al., 1990; Nys et al., 2004; Shakya et al., 2013; Singh et al., 2018).

In a recent study among children aged 1–14 years who were not previously exposed to antibiotics, Singh et al. (2018) found a close correlation between demographic factors and an increased incidence of commensal *E*. *coli* which were resistant to antibiotics. This highlights the overuse and misuse of antibiotics leading to a transmissible threat of multidrug resistance between commensals and pathogenic isolates (Singh et al., 2018). In another study by Infante et al. (2005), fecal samples tested for the presence of antibiotic resistance genes revealed a high prevalence of *sul*‐genes in commensal *E*. *coli* isolated from healthy children of Bolivia and Peru. This highlights the potential risks posed by commensal bacteria for the spread of antimicrobial resistance among pathogens (Infante et al., 2005). Antibiotic‐resistant *E. coli* (very high resistance to ampicillin and cotrimoxazole) isolated from the fecal microbiota of neonates less than a month of age, who had no previous history of antibiotic therapy, emphasize the rapidly growing threat of antibiotic resistance (Tule & Hassani, 2017). Analysis of the rates of transmission of antibiotic resistance between commensal bacteria and pathogenic members of Enterobacteriaceae (*E*. *coli* O157, and *Salmonella* spp) has also revealed that members of the Enterobacteriaceae family are capable of exchange, transfer, and dissemination of resistant genes from commensal microbiota to zoonotic pathogens and vice versa (Blake et al., 2003).

### 4.5 Clinical settings and surroundings

Clinical settings/hospitals have the highest level of antimicrobial consumption and hence are considered hotspots of AMR. Hospital effluents are expected to harbor a high number of resistant bacteria and genes. It was found that the effluent of Indian hospitals had high enough antibiotic concentrations to cause genotoxic modifications in bacterial strains (Diwan et al., 2010). Mutiyar and Mittal (2014) reported the dangerous levels of antibiotic residues (fluoroquinolones, sulfonamides, and tinidazoles) recovered from one of the hospital effluents in India (Mutiyar & Mittal, 2014). Although 80%–85% of antimicrobial residues in the hospital effluents can be removed efficiently by proper wastewater treatment (Duong et al., 2008), less than 45% of wastewater treatment systems work efficiently among all the healthcare facilities in India (Taneja & Sharma, 2019; World Health Organization, 2015).

Studies have shown that clinically relevant antibiotic resistance genes such as cefotaxime‐hydrolyzing beta‐lactamase (*blaCTX‐M*), *quinolone resistance determinant (qnrA)*, and New Delhi metallo‐beta‐lactamase (*blaNDM)* originating in the environmental bacteria *Kluyvera spp*., *Shewanella* *algae*, and *Erythrobacter litoralis*, respectively (Berendonk et al., 2015; Nordmann & Poirel, 2005; Oliver et al., 2001; Zheng et al., 2011), contribute to the challenge of effectively treating bacterial infections. However, anthropocentrism (a philosophical viewpoint that humans are the most important forms of life) has led to the view that these genes have evolved as antibiotic resistance genes, naturally (Walsh, 2013). The clinical importance of the soil resistome is that we know there can be an interchangeable transfer of resistance between the soil bacteria and pathogens, irrespective of whether resistance genes are moving from the clinic to the soil or vice versa (Forsberg et al., 2012). Cycoń et al. (2019) previously described alterations in the metabolizing capability of microorganisms in the soil present with antibiotics; namely, altered ability to metabolize different sources of carbon, and also their enzyme activity. The antibiotics also affected microbial biodiversity—biomass and the abundance of different categories; namely, gram‐positive bacteria, gram‐negative bacteria, and fungi in the microbial population. Exploring the array of antibiotic resistance genes in the soil had led to the discovery of new enzymes and genes which contribute to the emergence of antibiotic resistance in bacteria. The impact of antibiotics on microbial activity and biodiversity remains a great challenge (Cycoń et al., 2019).

Several examples in the literature have provided evidence for the transfer of antibiotic‐resistant genes in clinical environments via horizontal gene transfer. For example, Lerminiaux and Cameron (2019) concluded conjugation is the primary mode of horizontal gene transfer in clinical settings. Their review also offered that conditions for the spread of resistance genes via natural transformation and transduction existed in clinical environments (Lerminiaux & Cameron, 2019). Kac et al. (2004) conducted a study in a 17‐bed cardiac surgery intensive care unit in a large university teaching hospital to analyze the environmental contamination by extended‐spectrum ꞵ‐lactamase producing Enterobacteriaceae and compare the clinical and environmental strains. Molecular analysis revealed four species (three *Klebsiella oxytoca* and one *Enterobacter cloacae*) to be identical or having close similarities between clinical and environmental strains (Kac et al., 2004).

Another study has provided evidence for the exchange of antibiotic resistance genes between the bacteria in the environment and clinical pathogens (Forsberg et al., 2012). Most of the clinical pathogens belonged to be Proteobacteria (Rizzatti et al., 2017) and were cultured from the soil. Forsberg et al. (2012) described the presence of resistance gene cassettes in multidrug‐resistant soil bacteria. Gene cassettes against five classes of antibiotics: β‐lactams, amphenicols, aminoglycosides, tetracyclines, and sulfonamides were identified, which were present in clusters and shared identical nucleotide sequences with clinical pathogens. Mobile genetic elements flanked these gene clusters, suggesting they were acquired via horizontal gene transfer (Forsberg et al., 2012). Though studies of HGT in environments like wastewater treatment plants have progressed, no studies have identified and quantified the rate and drivers of HGT in clinical environments. Hence, there is a need to track these factors in clinical environments, where pathogens exacerbate the problem (Lerminiaux & Cameron, 2019).

## 5 CONCLUSION

The drivers and hotspots of antibiotic resistance together increase the burden of the spread of resistance determinants. There is currently a paucity of information regarding the impact of antibiotics on environmental microorganisms and the spread of resistance from clinical settings to the environment and vice versa. Soil harbors many microorganisms which are the primary source of antibiotics. It is, therefore, important to bridge the gap of scientific knowledge on these environmental microorganisms—specifically bacteria—habituated in the soil around clinical sites where many antibiotics are prescribed for medicinal use. The assessment of indices such as soil temperature, pH and moisture, and determination of resistant genes present in organisms isolated from the soil surrounding clinical settings over time, will help us understand the evolution of microorganisms and the impact of antibiotics on these organisms in clinical settings. Exploring these research gaps may further help us tackle the problem of antibiotic resistance which is considered one of the biggest threats to public health today.

## CONFLICT OF INTEREST

None declared.

## AUTHOR CONTRIBUTIONS

**Shalini Kunhikannan:** Conceptualization (equal); Writing‐original draft (lead). **Colleen J. Thomas:** Conceptualization (equal); Supervision (equal); Writing‐review & editing (equal). **Ashley E. Franks:** Conceptualization (equal); Project administration (equal); Supervision (equal); Writing‐review & editing (equal). **Sumana Mahadevaiah:** Conceptualization (equal); Supervision (equal); Writing‐review & editing (equal). **Sumana Kumar:** Conceptualization (equal); Supervision (equal); Writing‐review & editing (equal). **Steve Petrovski:** Conceptualization (equal); Project administration (equal); Supervision (equal); Writing‐review & editing (equal).

## ETHICS STATEMENT

None required.

## Data Availability

Not applicable.

## References

[mbo31197-bib-0001] Alekshun, M. N. , & Levy, S. B. (2007). Molecular mechanisms of antibacterial multidrug resistance. Cell, 128, 1037–1050. 17382878 10.1016/j.cell.2007.03.004

[mbo31197-bib-0002] Ayukekbong, J. A. , Ntemgwa, M. , & Atabe, A. N. (2017). The threat of antimicrobial resistance in developing countries: Causes and control strategies. Antimicrobial Resistance & Infection Control, 6, 47. 28515903 10.1186/s13756-017-0208-x PMC5433038

[mbo31197-bib-0003] Barber, M. (1947). Staphylococcal infection due to penicillin‐resistant strains. British Medical Journal, 2, 863. 20272443 10.1136/bmj.2.4534.863 PMC2056216

[mbo31197-bib-0004] Bartoloni, A. , Pallecchi, L. , Benedetti, M. , Fernandez, C. , Vallejos, Y. , Guzman, E. , Villagran, A. L. , Mantella, A. , Lucchetti, C. , Bartalesi, F. , Strohmeyer, M. , Bechini, A. , Gamboa, H. , Rodriguez, H. , Falkenberg, T. , Kronvall, G. , Gotuzzo, E. , Paradisi, F. , & Rossolini, G. M. (2006). Multidrug‐resistant commensal *Escherichia coli* in children, Peru and Bolivia. Emerging Infectious Diseases, 12, 907. 16707045 10.3201/eid1206.051258 PMC3373029

[mbo31197-bib-0005] Bengtsson‐Palme, J. , Kristiansson, E. , & Larsson, D. J. (2018). Environmental factors influencing the development and spread of antibiotic resistance. FEMS Microbiology Reviews, 42, fux053. 10.1093/femsre/fux053 PMC5812547 29069382

[mbo31197-bib-0006] Berendonk, T. U. , Manaia, C. M. , Merlin, C. , Fatta‐Kassinos, D. , Cytryn, E. , Walsh, F. , Bürgmann, H. , Sørum, H. , Norström, M. , & Pons, M.‐N. (2015). Tackling antibiotic resistance: The environmental framework. Nature Reviews Microbiology, 13, 310. 25817583 10.1038/nrmicro3439

[mbo31197-bib-0007] Berendsen, B. J. , Wegh, R. S. , Memelink, J. , Zuidema, T. , & Stolker, L. A. (2015). The analysis of animal faeces as a tool to monitor antibiotic usage. Talanta, 132, 258–268. 25476307 10.1016/j.talanta.2014.09.022

[mbo31197-bib-0008] Bhullar, K. , Waglechner, N. , Pawlowski, A. , Koteva, K. , Banks, E. D. , Johnston, M. D. , Barton, H. A. , & Wright, G. D. (2012). Antibiotic resistance is prevalent in an isolated cave microbiome. PLoS One, 7, e34953. 22509370 10.1371/journal.pone.0034953 PMC3324550

[mbo31197-bib-0009] Blake, D. , Hillman, K. , Fenlon, D. , & Low, J. (2003). Transfer of antibiotic resistance between commensal and pathogenic members of the Enterobacteriaceae under ileal conditions. Journal of Applied Microbiology, 95, 428–436. 12911689 10.1046/j.1365-2672.2003.01988.x

[mbo31197-bib-0010] Brady, N. C. , Weil, R. R. , & Weil, R. R. (2008). The nature and properties of soils. Prentice Hall.

[mbo31197-bib-0011] Cacace, D. , Fatta‐Kassinos, D. , Manaia, C. M. , Cytryn, E. , Kreuzinger, N. , Rizzo, L. , Karaolia, P. , Schwartz, T. , Alexander, J. , Merlin, C. , Garelick, H. , Schmitt, H. , de Vries, D. , Schwermer, C. U. , Meric, S. , Ozkal, C. B. , Pons, M.‐N. , Kneis, D. , & Berendonk, T. U. (2019). Antibiotic resistance genes in treated wastewater and in the receiving water bodies: A pan‐European survey of urban settings. Water Research, 162, 320–330. 31288142 10.1016/j.watres.2019.06.039

[mbo31197-bib-0012] Choudhury, R. , Panda, S. , & Singh, D. (2012). Emergence and dissemination of antibiotic resistance: A global problem. Indian Journal of Medical Microbiology, 30, 384. 23183460 10.4103/0255-0857.103756

[mbo31197-bib-0013] Christensen, B. B. , Sternberg, C. , Andersen, J. B. , Eberl, L. , Møller, S. , Givskov, M. , & Molin, S. (1998). Establishment of new genetic traits in a microbial biofilm community. Applied and Environment Microbiology, 64, 2247–2255. 10.1128/aem.64.6.2247-2255.1998 PMC106307 9603843

[mbo31197-bib-0014] Colodner, R. , Rock, W. , Chazan, B. , Keller, N. , Guy, N. , Sakran, W. , & Raz, R. (2004). Risk factors for the development of extended‐spectrum beta‐lactamase‐producing bacteria in nonhospitalized patients. European Journal of Clinical Microbiology and Infectious Diseases, 23, 163–167. 14986159 10.1007/s10096-003-1084-2

[mbo31197-bib-0015] Crofts, T. S. , Gasparrini, A. J. , & Dantas, G. (2017). Next‐generation approaches to understand and combat the antibiotic resistome. Nature Reviews Microbiology, 15, 422. 28392565 10.1038/nrmicro.2017.28 PMC5681478

[mbo31197-bib-0016] Cycoń, M. , Mrozik, A. , & Piotrowska‐Seget, Z. (2019). Antibiotics in the soil environment—Degradation and their impact on microbial activity and diversity. Frontiers in Microbiology, 10,338‐1–338‐45. 30906284 10.3389/fmicb.2019.00338 PMC6418018

[mbo31197-bib-0017] Czekalski, N. , Díez, E. G. , & Bürgmann, H. (2014). Wastewater as a point source of antibiotic‐resistance genes in the sediment of a freshwater lake. The ISME Journal, 8, 1381. 24599073 10.1038/ismej.2014.8 PMC4069405

[mbo31197-bib-0018] Daghrir, R. , & Drogui, P. (2013). Tetracycline antibiotics in the environment: A review. Environmental Chemistry Letters, 11, 209–227.

[mbo31197-bib-0019] Davies, J. (2012). Antibiotic discovery: Then and now. Microbiology Today, 39, 200–203.

[mbo31197-bib-0020] Davies, J. (2014). Antibiotic resistance and the golden age of microbiology. Upsala Journal of Medical Sciences, 119, 65–67. 24836049 10.3109/03009734.2014.898718 PMC4034562

[mbo31197-bib-0021] D'Costa, V. M. , McGrann, K. M. , Hughes, D. W. , & Wright, G. D. (2006). Sampling the antibiotic resistome. Science, 311, 374–377. 16424339 10.1126/science.1120800

[mbo31197-bib-0022] di Cesare, A. , Eckert, E. M. , D'Urso, S. , Bertoni, R. , Gillan, D. C. , Wattiez, R. , & Corno, G. (2016). Co‐occurrence of integrase 1, antibiotic and heavy metal resistance genes in municipal wastewater treatment plants. Water Research, 94, 208–214. 26945964 10.1016/j.watres.2016.02.049

[mbo31197-bib-0023] Diwan, V. , Tamhankar, A. J. , Khandal, R. K. , Sen, S. , Aggarwal, M. , Marothi, Y. , Iyer, R. V. , Sundblad‐Tonderski, K. , & Stålsby‐Lundborg, C. (2010). Antibiotics and antibiotic‐resistant bacteria in waters associated with a hospital in Ujjain, India. BMC Public Health, 10, 1–8. 20626873 10.1186/1471-2458-10-414 PMC2912816

[mbo31197-bib-0024] Duong, H. A. , Pham, N. H. , Nguyen, H. T. , Hoang, T. T. , Pham, H. V. , Pham, V. C. , Berg, M. , Giger, W. , & Alder, A. C. (2008). Occurrence, fate and antibiotic resistance of fluoroquinolone antibacterials in hospital wastewaters in Hanoi, Vietnam. Chemosphere, 72, 968–973. 18485444 10.1016/j.chemosphere.2008.03.009

[mbo31197-bib-0025] Elbossaty, W. (2017). Antibiotic drugs and multidrug resistance bacteria. International Journal of Public Health Safe, 2(3), 1–3.

[mbo31197-bib-0026] Emamalipour, M. , Seidi, K. , Zununi Vahed, S. , Jahanban‐Esfahlan, A. , Jaymand, M. , Majdi, H. , Amoozgar, Z. , Chitkushev, L. T. , Javaheri, T. , Jahanban‐Esfahlan, R. , & Zare, P. (2020). Horizontal gene transfer: From evolutionary flexibility to disease progression. Frontiers in Cell and Developmental Biology, 8,1–16. 32509768 10.3389/fcell.2020.00229 PMC7248198

[mbo31197-bib-0027] Evans, D. R. , Griffith, M. P. , Sundermann, A. J. , Shutt, K. A. , Saul, M. I. , Mustapha, M. M. , Marsh, J. W. , Cooper, V. S. , Harrison, L. H. , & van Tyne, D. (2020). Systematic detection of horizontal gene transfer across genera among multidrug‐resistant bacteria in a single hospital. eLife, 9, e53886. 32285801 10.7554/eLife.53886 PMC7156236

[mbo31197-bib-0028] Fick, J. , Söderström, H. , Lindberg, R. H. , Phan, C. , Tysklind, M. , & Larsson, D. J. (2009). Contamination of surface, ground, and drinking water from pharmaceutical production. Environmental Toxicology and Chemistry, 28, 2522–2527. 19449981 10.1897/09-073.1

[mbo31197-bib-0029] Finley, R. L. , Collignon, P. , Larsson, D. J. , McEwen, S. A. , Li, X.‐Z. , Gaze, W. H. , Reid‐Smith, R. , Timinouni, M. , Graham, D. W. , & Topp, E. (2013). The scourge of antibiotic resistance: the important role of the environment. Clinical Infectious Diseases, 57, 704–710. 23723195 10.1093/cid/cit355

[mbo31197-bib-0030] Forsberg, K. J. , Reyes, A. , Wang, B. , Selleck, E. M. , Sommer, M. O. , & Dantas, G. (2012). The shared antibiotic resistome of soil bacteria and human pathogens. Science, 337, 1107–1111. 22936781 10.1126/science.1220761 PMC4070369

[mbo31197-bib-0031] Gao, P. , Munir, M. , & Xagoraraki, I. (2012). Correlation of tetracycline and sulfonamide antibiotics with corresponding resistance genes and resistant bacteria in a conventional municipal wastewater treatment plant. Science of the Total Environment, 421, 173–183. 10.1016/j.scitotenv.2012.01.061 22369865

[mbo31197-bib-0032] Gashaw, M. , Berhane, M. , Bekele, S. , Kibru, G. , Teshager, L. , Yilma, Y. , Ahmed, Y. , Fentahun, N. , Assefa, H. , & Wieser, A. (2018). Emergence of high drug resistant bacterial isolates from patients with health care associated infections at Jimma University medical center: A cross sectional study. Antimicrobial Resistance & Infection Control, 7, 138. 30479751 10.1186/s13756-018-0431-0 PMC6245755

[mbo31197-bib-0033] Gelband, H. , Miller‐Petrie, M. , Pant, S. , Gandra, S. , Levinson, J. , Barter, D. , White, A. , & Laxminarayan, R. (2015). State of the world’s antibiotics, 2015. Center for Disease Dynamics, Economics & Policy.

[mbo31197-bib-0034] Gibbons, A. (2015). Resistance to antibiotics found in isolated Amazonian tribe. Science. 10.1126/science.aab2509

[mbo31197-bib-0035] Gogarten, J. P. , & Townsend, J. P. (2005). Horizontal gene transfer, genome innovation and evolution. Nature Reviews Microbiology, 3, 679–687. 16138096 10.1038/nrmicro1204

[mbo31197-bib-0036] Gullberg, E. , Cao, S. , Berg, O. G. , Ilbäck, C. , Sandegren, L. , Hughes, D. , & Andersson, D. I. (2011). Selection of resistant bacteria at very low antibiotic concentrations. PLoS Pathogens, 7, e1002158. 21811410 10.1371/journal.ppat.1002158 PMC3141051

[mbo31197-bib-0037] Harbarth, S. , Theuretzbacher, U. , Hackett, J. , DRIVE‐AB Consortium , Adriaenssens, N. , Anderson, J. , Antonisse, A. , Årdal, C. , Baillon‐Plot, N. , & Baraldi, E. (2015). Antibiotic research and development: Business as usual? Journal of Antimicrobial Chemotherapy, 70, 1604–1607. 10.1093/jac/dkv020 25673635

[mbo31197-bib-0038] Hutchings, M. I. , Truman, A. W. , & Wilkinson, B. (2019). Antibiotics: Past, present and future. Current Opinion in Microbiology, 51, 72–80. 31733401 10.1016/j.mib.2019.10.008

[mbo31197-bib-0039] Infante, B. , Grape, M. , Larsson, M. , Kristiansson, C. , Pallecchi, L. , Rossolini, G. M. , & Kronvall, G. (2005). Acquired sulphonamide resistance genes in faecal *Escherichia coli* from healthy children in Bolivia and Peru. International Journal of Antimicrobial Agents, 25, 308–312. 15784310 10.1016/j.ijantimicag.2004.12.004

[mbo31197-bib-0040] Jain, R. , Rivera, M. C. , Moore, J. E. , & Lake, J. A. (2003). Horizontal gene transfer accelerates genome innovation and evolution. Molecular Biology and Evolution, 20, 1598–1602. 12777514 10.1093/molbev/msg154

[mbo31197-bib-0041] Jeśman, C. , Młudzik, A. , & Cybulska, M. (2011). History of antibiotics and sulphonamides discoveries. Polski Merkuriusz Lekarski: Organ Polskiego Towarzystwa Lekarskiego, 30, 320–322. 21675132

[mbo31197-bib-0042] Jiang, S. C. , & Paul, J. H. (1998). Gene transfer by transduction in the marine environment. Applied and Environmental Microbiology, 64, 2780. 9687430 10.1128/aem.64.8.2780-2787.1998 PMC106772

[mbo31197-bib-0043] Kac, G. , Podglajen, I. , Vaupré, S. , Colardelle, N. , Buu‐Hoï, A. , & Gutmann, L. (2004). Molecular epidemiology of extended‐spectrum beta‐lactamase–producing enterobacteriaceae isolated from environmental and clinical specimens in a cardiac surgery intensive care unit. Infection Control and Hospital Epidemiology, 25, 852–855. 15518028 10.1086/502308

[mbo31197-bib-0044] Karkman, A. , Do, T. T. , Walsh, F. , & Virta, M. P. (2018). Antibiotic‐resistance genes in waste water. Trends in Microbiology, 26, 220–228. 29033338 10.1016/j.tim.2017.09.005

[mbo31197-bib-0045] Katale, B. Z. , Misinzo, G. , Mshana, S. E. , Chiyangi, H. , Campino, S. , Clark, T. G. , Good, L. , Rweyemamu, M. M. , & Matee, M. I. (2020). Genetic diversity and risk factors for the transmission of antimicrobial resistance across human, animals and environmental compartments in East Africa: A review. Antimicrobial Resistance & Infection Control, 9, 1–20. 32762743 10.1186/s13756-020-00786-7 PMC7409632

[mbo31197-bib-0046] Khan, A. , & Rao, T. S. (2019). Molecular evolution of xenobiotic degrading genes and mobile DNA elements in soil bacteria. In Surajit Das & Hirak Dash Microbial diversity in the genomic era (pp. 657–678). Elsevier.

[mbo31197-bib-0047] Khan, F. A. , Söderquist, B. , & Jass, J. (2019). Prevalence and diversity of antibiotic resistance genes in Swedish aquatic environments impacted by household and hospital wastewater. Frontiers in Microbiology, 10, 688. 31019498 10.3389/fmicb.2019.00688 PMC6458280

[mbo31197-bib-0048] Knöppel, A. , Näsvall, J. , & Andersson, D. I. (2017). Evolution of antibiotic resistance without antibiotic exposure. Antimicrobial Agents and Chemotherapy, 61(11), e01495‐17 28893783 10.1128/AAC.01495-17 PMC5655081

[mbo31197-bib-0049] Kruse, H. , & Sørum, H. (1994). Transfer of multiple drug resistance plasmids between bacteria of diverse origins in natural microenvironments. Applied and Environmental Microbiology, 60, 4015–4021. 11865872 10.1128/aem.60.11.4015-4021.1994 PMC201930

[mbo31197-bib-0050] Kumar, P. , Bag, S. , Ghosh, T. S. , Dey, P. , Dayal, M. , Saha, B. , Verma, J. , Pant, A. , Saxena, S. , & Desigamani, A. (2017). Molecular insights into antimicrobial resistance traits of multidrug resistant enteric pathogens isolated from India. Scientific Reports, 7, 1–12. 29089611 10.1038/s41598-017-14791-1 PMC5663842

[mbo31197-bib-0051] Lacroix, B. , & Citovsky, V. (2016). Transfer of DNA from bacteria to eukaryotes. mBio, 7, e00863‐16. 27406565 10.1128/mBio.00863-16 PMC4958254

[mbo31197-bib-0052] Lang, A. S. , Zhaxybayeva, O. , & Beatty, J. T. (2012). Gene transfer agents: Phage‐like elements of genetic exchange. Nature Reviews Microbiology, 10, 472–482. 22683880 10.1038/nrmicro2802 PMC3626599

[mbo31197-bib-0053] Lapara, T. M. , Burch, T. R. , McNamara, P. J. , Tan, D. T. , Yan, M. , & Eichmiller, J. J. (2011). Tertiary‐treated municipal wastewater is a significant point source of antibiotic resistance genes into Duluth‐Superior Harbor. Environmental Science & Technology, 45, 9543–9549. 21981654 10.1021/es202775r

[mbo31197-bib-0054] Lerminiaux, N. A. , & Cameron, A. D. (2019). Horizontal transfer of antibiotic resistance genes in clinical environments. Canadian Journal of Microbiology, 65, 34–44. 30248271 10.1139/cjm-2018-0275

[mbo31197-bib-0055] Lester, S. , Pla, M. , Wang, F. , Jiang, H. , & O'Brien, T. (1990). The carriage of *Escherichia coli* resistant to antimicrobial agents by healthy children in Boston, in Caracas, Venezuela, and in Qin Pu, China. New England Journal of Medicine, 323, 285–289. 10.1056/NEJM199008023230501 2195344

[mbo31197-bib-0056] Levy, S. B. , & Marshall, B. (2004). Antibacterial resistance worldwide: Causes, challenges and responses. Nature Medicine, 10, S122. 10.1038/nm1145 15577930

[mbo31197-bib-0057] Linton, K. , Richmond, M. , Bevan, R. , & Gillespie, W. (1974). Antibiotic resistance and R factors in coliform bacilli isolated from hospital and domestic sewage. Journal of Medical Microbiology, 7, 91–103. 4604101 10.1099/00222615-7-1-91

[mbo31197-bib-0058] Macuch, P. , Seckarova, A. , Parrakova, E. , Krcmery, V. , & Vymola, F. (1967). Transfer of tetracycline resistance from *Escherichia coli* to other enterobacteriaceae in vitro. Zeitschrift für Allgemeine Mikrobiologie, 7, 159–162. 4876865 10.1002/jobm.3630070212

[mbo31197-bib-0059] Marathe, N. P. , Regina, V. R. , Walujkar, S. A. , Charan, S. S. , Moore, E. R. , Larsson, D. J. , & Shouche, Y. S. (2013). A treatment plant receiving waste water from multiple bulk drug manufacturers is a reservoir for highly multi‐drug resistant integron‐bearing bacteria. PLoS One, 8, e77310. 24204801 10.1371/journal.pone.0077310 PMC3812170

[mbo31197-bib-0060] Michael, C. A. , Dominey‐Howes, D. , & Labbate, M. (2014). The antimicrobial resistance crisis: Causes, consequences, and management. Frontiers in Public Health, 2, 145. 25279369 10.3389/fpubh.2014.00145 PMC4165128

[mbo31197-bib-0061] Munita, J. M. , & Arias, C. A. (2016). Mechanisms of antibiotic resistance. Microbiology Spectrum, 4(2), 1–37. 10.1128/microbiolspec.VMBF-0016-2015 PMC4888801 27227291

[mbo31197-bib-0062] Mutiyar, P. K. , & Mittal, A. K. (2014). Risk assessment of antibiotic residues in different water matrices in India: Key issues and challenges. Environmental Science and Pollution Research, 21, 7723–7736. 24627199 10.1007/s11356-014-2702-5

[mbo31197-bib-0063] Nathan, C. (2004). Antibiotics at the crossroads. Nature, 431, 899. 15496893 10.1038/431899a

[mbo31197-bib-0064] Nathan, C. , & Cars, O. (2014). Antibiotic resistance—Problems, progress, and prospects. New England Journal of Medicine, 371, 1761–1763. 10.1056/NEJMp1408040 25271470

[mbo31197-bib-0065] Nikaido, H. (2009). Multidrug resistance in bacteria. Annual Review of Biochemistry, 78, 119–146. 10.1146/annurev.biochem.78.082907.145923 PMC2839888 19231985

[mbo31197-bib-0066] Nordmann, P. , & Poirel, L. (2005). Emergence of plasmid‐mediated resistance to quinolones in Enterobacteriaceae. Journal of Antimicrobial Chemotherapy, 56, 463–469. 10.1093/jac/dki245 16020539

[mbo31197-bib-0067] Nys, S. , Okeke, I. N. , Kariuki, S. , Dinant, G. J. , Driessen, C. , & Stobberingh, E. E. (2004). Antibiotic resistance of faecal *Escherichia coli* from healthy volunteers from eight developing countries. Journal of Antimicrobial Chemotherapy, 54, 952–955. 10.1093/jac/dkh448 15471998

[mbo31197-bib-0068] Ochman, H. , Lawrence, J. G. , & Groisman, E. A. (2000). Lateral gene transfer and the nature of bacterial innovation. Nature, 405, 299–304. 10830951 10.1038/35012500

[mbo31197-bib-0069] Oliver, A. , Pérez‐Díaz, J. C. , Coque, T. M. , Baquero, F. , & Cantón, R. (2001). Nucleotide sequence and characterization of a novel cefotaxime‐hydrolyzing β‐lactamase (CTX‐M‐10) isolated in Spain. Antimicrobial Agents and Chemotherapy, 45, 616–620. 11158766 10.1128/AAC.45.2.616-620.2001 PMC90338

[mbo31197-bib-0070] O'Neill, J. (2016). Tackling drug‐resistant infections globally: Final report and recommendations.

[mbo31197-bib-0071] Price, V. J. , McBride, S. W. , Hullahalli, K. , Chatterjee, A. , Duerkop, B. A. , & Palmer, K. L. (2019). *Enterococcus faecalis* CRISPR‐Cas is a robust barrier to conjugative antibiotic resistance dissemination in the murine intestine. mSphere, 4(4), e00464‐19. 31341074 10.1128/mSphere.00464-19 PMC6656873

[mbo31197-bib-0072] Redondo‐Salvo, S. , Fernández‐López, R. , Ruiz, R. , Vielva, L. , de Toro, M. , Rocha, E. P. C. , Garcillán‐Barcia, M. P. , & de la Cruz, F. (2020). Pathways for horizontal gene transfer in bacteria revealed by a global map of their plasmids. Nature Communications, 11, 3602. 10.1038/s41467-020-17278-2 PMC7367871 32681114

[mbo31197-bib-0073] Rizzatti, G. , Lopetuso, L. R. , Gibiino, G. , Binda, C. , & Gasbarrini, A. (2017). Proteobacteria: A common factor in human diseases. BioMed Research International, 2017, 9351507. 29230419 10.1155/2017/9351507 PMC5688358

[mbo31197-bib-0074] Rizzo, L. , Manaia, C. , Merlin, C. , Schwartz, T. , Dagot, C. , Ploy, M. , Michael, I. , & Fatta‐Kassinos, D. (2013). Urban wastewater treatment plants as hotspots for antibiotic resistant bacteria and genes spread into the environment: A review. Science of the Total Environment, 447, 345–360. 10.1016/j.scitotenv.2013.01.032 23396083

[mbo31197-bib-0075] Sahoo, K. C. , Tamhankar, A. J. , Johansson, E. , & Lundborg, C. S. (2010). Antibiotic use, resistance development and environmental factors: A qualitative study among healthcare professionals in Orissa, India. BMC Public Health, 10, 629. 20964815 10.1186/1471-2458-10-629 PMC2973940

[mbo31197-bib-0076] Salyers, A. A. , Gupta, A. , & Wang, Y. (2004). Human intestinal bacteria as reservoirs for antibiotic resistance genes. Trends in Microbiology, 12, 412–416. 15337162 10.1016/j.tim.2004.07.004

[mbo31197-bib-0077] Sarmah, A. K. , Meyer, M. T. , & Boxall, A. B. (2006). A global perspective on the use, sales, exposure pathways, occurrence, fate and effects of veterinary antibiotics (VAs) in the environment. Chemosphere, 65, 725–759. 16677683 10.1016/j.chemosphere.2006.03.026

[mbo31197-bib-0078] Shakya, P. , Barrett, P. , Diwan, V. , Marothi, Y. , Shah, H. , Chhari, N. , Tamhankar, A. J. , Pathak, A. , & Lundborg, C. S. (2013). Antibiotic resistance among *Escherichia coli* isolates from stool samples of children aged 3 to 14 years from Ujjain, India. BMC Infectious Diseases, 13, 477. 24124728 10.1186/1471-2334-13-477 PMC3853101

[mbo31197-bib-0079] Singh, A. K. , Das, S. , Singh, S. , Gajamer, V. R. , Pradhan, N. , Lepcha, Y. D. , & Tiwari, H. K. (2018). Prevalence of antibiotic resistance in commensal *Escherichia coli* among the children in rural hill communities of Northeast India. PLoS One, 13, e0199179. 29912980 10.1371/journal.pone.0199179 PMC6005495

[mbo31197-bib-0080] Smillie, C. S. , Smith, M. B. , Friedman, J. , Cordero, O. X. , David, L. A. , & Alm, E. J. (2011). Ecology drives a global network of gene exchange connecting the human microbiome. Nature, 480, 241–244. 22037308 10.1038/nature10571

[mbo31197-bib-0081] Solioz, M. , & Marrs, B. (1977). The gene transfer agent of *Rhodopseudomonas capsulata*: Purification and characterization of its nucleic acid. Archives of Biochemistry and Biophysics, 181, 300–307. 879805 10.1016/0003-9861(77)90508-2

[mbo31197-bib-0082] Soucy, S. M. , Huang, J. , & Gogarten, J. P. (2015). Horizontal gene transfer: Building the web of life. Nature Reviews Genetics, 16, 472–482. 10.1038/nrg3962 26184597

[mbo31197-bib-0083] Swift, B. M. , Bennett, M. , Waller, K. , Dodd, C. , Murray, A. , Gomes, R. L. , Humphreys, B. , Hobman, J. L. , Jones, M. A. , & Whitlock, S. E. (2019). Anthropogenic environmental drivers of antimicrobial resistance in wildlife. Science of the Total Environment, 649, 12–20. 10.1016/j.scitotenv.2018.08.180 30170212

[mbo31197-bib-0084] Takeuchi, N. , Kaneko, K. , & Koonin, E. V. (2014). Horizontal gene transfer can rescue prokaryotes from Muller’s ratchet: Benefit of DNA from dead cells and population subdivision. G3: Genes, Genomes, Genetics, 4, 325–339. 24347631 10.1534/g3.113.009845 PMC3931566

[mbo31197-bib-0085] Taneja, N. , & Sharma, M. (2019). Antimicrobial resistance in the environment: The Indian scenario. The Indian Journal of Medical Research, 149, 119. 31219076 10.4103/ijmr.IJMR_331_18 PMC6563737

[mbo31197-bib-0086] Tanwar, J. , Das, S. , Fatima, Z. , & Hameed, S. (2014). Multidrug resistance: An emerging crisis. Interdisciplinary Perspectives on Infectious Diseases, 2014,541340. 25140175 10.1155/2014/541340 PMC4124702

[mbo31197-bib-0087] Tule, A. , & Hassani, U. (2017). Colonization with antibiotic‐resistant *E. coli* in commensal fecal flora of newborns. International Journal of Current Microbiology and Applied Science, 6, 1623–1629.

[mbo31197-bib-0088] van Dijk, B. , Hogeweg, P. , Doekes, H. M. , & Takeuchi, N. (2020). Slightly beneficial genes are retained by bacteria evolving DNA uptake despite selfish elements. eLife, 9, e56801. 32432548 10.7554/eLife.56801 PMC7316506

[mbo31197-bib-0089] Walsh, F. (2013). Investigating antibiotic resistance in non‐clinical environments. Frontiers in Microbiology, 4, 19. 23423602 10.3389/fmicb.2013.00019 PMC3573686

[mbo31197-bib-0090] Wang, F.‐H. , Qiao, M. , Su, J.‐Q. , Chen, Z. , Zhou, X. , & Zhu, Y.‐G. (2014). High throughput profiling of antibiotic resistance genes in urban park soils with reclaimed water irrigation. Environmental Science & Technology, 48, 9079–9085. 25057898 10.1021/es502615e

[mbo31197-bib-0091] Wellington, E. M. , Boxall, A. B. , Cross, P. , Feil, E. J. , Gaze, W. H. , Hawkey, P. M. , Johnson‐Rollings, A. S. , Jones, D. L. , Lee, N. M. , & Otten, W. (2013). The role of the natural environment in the emergence of antibiotic resistance in Gram‐negative bacteria. The Lancet Infectious Diseases, 13, 155–165. 23347633 10.1016/S1473-3099(12)70317-1

[mbo31197-bib-0092] Wichmann, F. , Udikovic‐Kolic, N. , Andrew, S. , & Handelsman, J. (2014). Diverse antibiotic resistance genes in dairy cow manure. mBio, 5,e01017‐13. 10.1128/mBio.01017-13 PMC3993861 24757214

[mbo31197-bib-0093] Wistrand‐Yuen, E. , Knopp, M. , Hjort, K. , Koskiniemi, S. , Berg, O. G. , & Andersson, D. I. (2018). Evolution of high‐level resistance during low‐level antibiotic exposure. Nature Communications, 9, 1–12. 10.1038/s41467-018-04059-1 PMC5913237 29686259

[mbo31197-bib-0094] World Health Organization . (2015). Water, sanitation and hygiene in health care facilities: Status in low and middle income countries and way forward.

[mbo31197-bib-0095] Xiong, W. , Sun, Y. , Ding, X. , Wang, M. , & Zeng, Z. (2015). Selective pressure of antibiotics on ARGs and bacterial communities in manure‐polluted freshwater‐sediment microcosms. Frontiers in Microbiology, 6, 194. 25814986 10.3389/fmicb.2015.00194 PMC4356103

[mbo31197-bib-0096] Xu, L. , Ouyang, W. , Qian, Y. , Su, C. , Su, J. , & Chen, H. (2016). High‐throughput profiling of antibiotic resistance genes in drinking water treatment plants and distribution systems. Environmental Pollution, 213, 119–126. 26890482 10.1016/j.envpol.2016.02.013

[mbo31197-bib-0097] Zheng, B. , Tan, S. , Gao, J. , Han, H. , Liu, J. , Lu, G. , Liu, D. , Yi, Y. , Zhu, B. , & Gao, G. F. (2011). An unexpected similarity between antibiotic‐resistant NDM‐1 and beta‐lactamase II from *Erythrobacter litoralis* . Protein & Cell, 2, 250–258. 21468894 10.1007/s13238-011-1027-0 PMC4875309

